# Complete Genome Sequences and Transmission Electron Micrographs of *Listeria* Phages of the Genus *Homburgvirus*

**DOI:** 10.1128/MRA.00825-19

**Published:** 2019-10-10

**Authors:** Lauren K. Hudson, Tracey L. Peters, Yaxiong Song, Thomas G. Denes

**Affiliations:** a Department of Food Science, The University of Tennessee, Knoxville, Tennessee, USA; Queens College

## Abstract

Bacteriophages that infect the foodborne pathogen Listeria monocytogenes were previously isolated from New York dairy farms. The complete genome sequences for three of these Listeria phages, with genome sizes of 64.6 to 65.7 kb, are presented here. *Listeria* phages LP-010, LP-013, and LP-031-2 are siphoviruses that belong to the genus Homburgvirus.

## ANNOUNCEMENT

Lytic bacteriophages can be used as a biocontrol agent targeting the foodborne bacterial pathogen Listeria monocytogenes in food or food processing environments (1–4). L. monocytogenes caused 116 laboratory-confirmed infections in the United States in 2015, with relatively high hospitalization and mortality rates compared to those caused by other foodborne pathogens (5, 6). Previously studied *Listeria* phages suitable for food-related biocontrol include Homburgvirus P70 (7). *Homburgvirus* phages have a unique morphology (flexible, noncontractile tails and elongated capsids, as seen in Enterococcus phages [8]) and improved lytic ability at lower temperatures (9).

Phages LP-010, LP-013, and LP-031, which infect L. monocytogenes, were previously isolated from dairy farm silage collected in New York (10). These were selected for sequencing because they exhibited activity against mutant L. monocytogenes strains that were resistant to most of our phage collection (11). LP-010 and LP-013 were isolated with L. monocytogenes strain FSL J1-208 and LP-031 with strain MACK (8, 10, 12, 13). All of the phages were propagated on MACK. DNA was extracted from purified phage stocks following a modified phenol-chloroform method (14). Libraries were prepared using Nextera XT kits and sequenced with an Illumina MiSeq platform using 300-bp paired-end read chemistry and 275 cycles. An average of 217,825 total reads per sample were acquired, and the average read length was 250 bp. Raw reads were trimmed with Trimmomatic v0.35 (ILLUMINACLIP:NexteraPE-PE.fa:2:30:10 LEADING:3 TRAILING:3 SLIDINGWINDOW:4:15 MINLEN:36) (15) and quality checked with FastQC v0.11.7 (16). Trimmed reads were assembled with SPAdes v3.12.0 (using defaults but with the careful setting) (17), and assembly statistics were generated using BBMap v38.08 (18), SAMtools v0.1.8 (19), and QUAST v4.6.3 (20). Assemblies were reoriented to start at the large terminase subunit and were then annotated using RAST*tk* (modifying pipeline to run “annotate-proteins-phage” before “annotate-proteins-kmer-v2”) (21). The read coverage across the newly formed contig junction, where the original contig ends were joined, was consistent with the rest of the assembly. This confirmed that the genomes are circularly permuted, which is consistent with other *Homburgvirus* phages (7, 8). Average nucleotide identity (ANI) between phages and *Homburgvirus* RefSeq assemblies was calculated with MUMmer (ANIm) using JSpeciesWS (22, 23).

**FIG 1:**
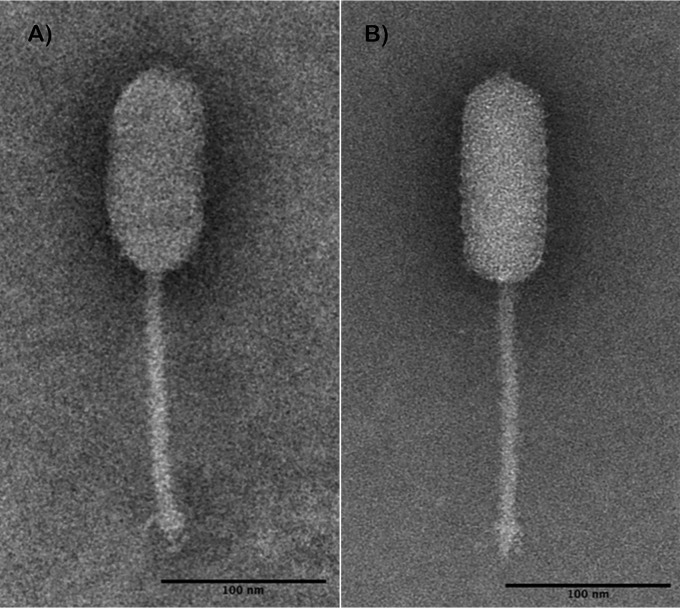
Transmission electron micrographs of LP-010 (A) and LP-013 (B). Transmission electron microscopy (TEM) was performed as previously described (24), with modifications. Phages were washed with 0.1 M ammonium acetate solution (pH 7), centrifuged at 21,000 × *g*, deposited onto 150- to 200-mesh carbon-coated Formvar film copper grids, and stained with 1% phosphotungstic acid (PTA; pH 7.4). Samples were imaged using a JEOL 1400 Flash transmission electron microscope at 80 kV with final magnifications of ×69,500 to ×111,200 and analyzed using Fiji 3 (25).

LP-031 assembled into two contigs (133.2 kb and 65.5 kb), which were redesignated LP-031-1 and LP-031-2, respectively. LP-031-1 was similar to Pecentumvirus phages and is not discussed here. LP-010, LP-013, and LP-031-2 have 64.6- to 65.7-kb circularly permuted genomes. The assemblies had 104× to 1,129× coverage and ∼36.4% G+C content and contained 108 to 114 coding sequences and no tRNAs. These three genomes have 97.65 to 99.35% ANIm across 95.80 to 99.58% of the aligned sequences. They are most similar to LP-114 (*Homburgvirus* genus), with 97.5 to 97.8% ANIm across 93.6 to 96.5% of the aligned sequences. LP-010 and LP-013 are *Siphoviridae* phages with elongated capsids measuring 66 by 133 nm and 58 by 129 nm, respectively, and tail lengths of 167 nm (Fig. 1).

### Data availability.

These phages are under BioProject number PRJNA544516 (BioSample numbers SAMN12053434, SAMN12053435, and SAMN12053437). Raw reads were deposited in the SRA (SRR9597079, SRR9597080, and SRR9597081) and the annotated genomes in GenBank (accession numbers MN114082, MN114083, and MN128593).
